# Hyaluronic Acid-like Skin Plumping and Radiance Benefits of a *Porphyridium* Sulfated Exopolysaccharide- and Natural PDRN-Rich Extract

**DOI:** 10.3390/md24030099

**Published:** 2026-03-01

**Authors:** Fabien Havas, Shlomo Krispin, Moshe Cohen, Joan Attia-Vigneau

**Affiliations:** 1 Lucas Meyer Cosmetics Israel, Faran 4, Yavne 8122503, Israel; fabien.havas@lucasmeyercosmetics.com (F.H.); shlomo.krispin@lucasmeyercosmetics.com (S.K.); moshe.cohen@lucasmeyercosmetics.com (M.C.); 2 Lucas Meyer Cosmetics France, 195 Route d’Espagne, 31036 Toulouse, France

**Keywords:** microalga, *Porphyridium*, exopolysaccharide, natural PDRN, hyaluronic acid, cosmetic, plumpness, radiance

## Abstract

Red microalga *Porphyridium cruentum* produces a sulfated exopolysaccharide (EPS), which enables its survival in challenging intertidal and spray zones. Extracellular polysaccharide hyaluronic acid (HA) plays important roles in skin hydration, elasticity, and volume. However, with aging, HA decreases and loses effectiveness, reducing skin moisture retention and firmness, and increasing signs of aging. An effective topical alternative to injectable HA replacement remains a largely unmet need. An extract of *Porphyridium* cultivated in natural sunlight, rich in EPS and polydeoxyribonucleotides (PDRNs), significantly activated the ADORA2A receptor in a CHO model, as well as reduced inflammation and increased collagen and HA production, autophagic flux, and key autophagy gene expression in dermal fibroblast cultures. In a double-blind clinical trial with placebo and HA benchmark controls, the *Porphyridium* extract delivered significant HA-like skin plumpness, hydration, and radiance benefits, and reduced signs of aging. The extract generally equaled or exceeded the HA benchmark. Its meaningful, swift HA-like activity shows potential for a safe, natural, and arguably more powerful HA-like alternative.

## 1. Introduction

Plump (or turgid) skin may be characterized as well-hydrated, firm, possessing a natural roundness and elasticity, and reflecting light in a manner associated with a radiant and youthful appearance—particularly in the facial area. Conversely, decreased perceived skin plumpness is associated with increased apparent age [1]. Preserving these skin characteristics requires, *inter alia*, maintaining levels of key extracellular matrix (ECM) components, including collagen, elastin, and hyaluronic acid (HA) in skin.

The collagen family of proteins, in particular collagen I, is a major component of conjunctive tissues strongly implicated in the skin’s biomechanical properties. Collagen I’s precursor pro-collagen I is synthesized by fibroblasts, and serves as a biomarker for collagen I synthesis. Starting in our late 20s, collagen production declines by about 1% to 1.5% annually [2]. The other major collagen type in skin is collagen III; with fine, flexible fibers, it is also found in the dermis, and also plays a part in skin elasticity and structural integrity, and in wound healing, where it regulates matrix architecture and mechanosensing [3,4]. Like for collagen I, the abundance of collagen III tends to decrease with age [5].

HA is a negatively charged glycosaminoglycan polysaccharide naturally synthesized in the skin, mainly by dermal fibroblasts, and composed of *D*-glucuronic acid and *N*-acetyl-D-glucosamine [6,7,8]. HA is produced in the skin in a range of molecular weights (MW), ranging from several kDa and up to 1200 kDa [9]. HA is a crucial component of the skin ECM, in particular in maintaining hydration by water binding based on its capacity to retain up to 1000 times its weight in water. Skin contains over half of the body’s HA, predominantly concentrated in the dermis [6,10]. HA is therefore widely recognized as essential for maintaining skin hydration, elasticity, and plumpness, contributing significantly to youthful skin appearance. HA also plays a role in tissue repair (wound healing), cell proliferation and migration, and the modulation of inflammatory processes [9,11]. High-molecular-weight HA possesses anti-inflammatory properties, but conversely, low-molecular-weight HA has been shown to promote pro-inflammatory processes [9,12]. HA also enhances collagen synthesis, contributing to ECM homeostasis [9,11]. Recently, hyaluronic acid has also been reported to upregulate autophagic flux and prevent cell entry into senescence [13]. Autophagy digests and recycles defective or damaged elements of cells or tissues, including through absorption into autophagosome vesicles and degradation by lysosome enzymes [14,15,16,17,18,19]. The resulting small-molecule products may be used in building new biological components or eliminated as waste. This removal of unneeded, dysfunctional, or harmful entities is a key part of healthy tissue homeostasis. In aging and some pathological states, autophagy may be adversely impacted, and tissues may lose the ability to properly eliminate harmful elements, leading to their accumulation and the impairment of overall tissue function. Autophagy has thus been a major focus in the investigation of skin aging and the development of technologies aiming to restore the skin’s biology to its youthful efficiency.

HA is naturally degraded in skin by hyaluronidase enzymes, with turnover rates as short as 24 h. Non-enzymatic processes involving reactive oxygen species (ROS) can accelerate HA breakdown [6,20]. Skin aging and exogenous insults such as UV exposure can dramatically reduce the synthesis of HA and the expression of HA synthase enzymes, while accelerating its degradation by hyaluronidases and oxidative processes—resulting in overall reduced HA content [6], which can be halved by the age of fifty. Remaining dermal HA can also show alterations in structure and functionality, including increased binding to tissue structures and a reduction in polymer size [6]. Clinical manifestations of reductions in HA levels and collagen can include losses of skin volume, elasticity, hydration, and radiance, and the development of wrinkles and fine lines [6].

As a result, the delivery of HA from cosmetic formulations as a replacement for lost endogenous HA has been widely employed as a strategy for restoring a youthful and plumped skin appearance, providing immediate hydration benefits and making skin appear smoother and fuller [6,21]. There is also a notable market for injectable HA; however, this carries the expected drawbacks of cost, inconvenience, discomfort, and possible additional side effects of repeated injections.

HA for use in topical formulations can be derived from animal sources (e.g., rooster combs) or non-animal sources (e.g., bacterial fermentation) [12]. As with endogenous HA in skin, commercial HA exists in various molecular weight ranges, with varying attendant benefits and drawbacks. High-molecular-weight HA remains on the skin surface, retaining moisture at the outermost layers. This can improve skin hydration and reduce the appearance of fine lines. Conversely, low-molecular-weight HA penetrates deeper into the skin, stimulating collagen production and promoting overall skin health [6,22,23,24]. By utilizing a combination of high- and low-molecular-weight HA fractions in topical products, cosmetic formulations seek to deliver both surface hydration and deeper structural improvements, leading to a more youthful and radiant appearance.

However, HA does have some limitations in this context. The required combination of molecular weight fractions to achieve the full desired effect can carry significant cost, especially given that low-molecular-weight HA grades tend to be highly expensive. Additionally, hyaluronic acid-based formulations can have poor degradation rates due to their sensitivity to hyaluronidase, and unfavorable mechanical properties (such as pH and temperature sensitivity), limiting their application [25,26]. 

Due to their evolutionary adaptation to various environmental conditions, marine organisms such as algae have acquired the ability to synthesize unique bioactive biopolymers, including sulfated polysaccharides [27,28]. Some of these possess strong water-binding capacity, reminiscent of high-molecular-weight HA’s ability to form a hydrating film on the skin’s surface. Additionally, some sulfated polysaccharides have exhibited antioxidant activity [29], as well as a potential ability to stimulate collagen synthesis. Thus, algal polysaccharides may represent valuable ingredients for formulating cosmetic products aimed at enhancing hydration, promoting a youthful appearance, and protecting the skin from environmental stressors [27,30]. Many cosmetic formulations use algal bioactive metabolites or algal cells as moisturizers, texture-enhancing agents, anti-wrinkle agents, whitening agents, sunscreens, anti-cellulite agents, thickening agents, and for hair care [30,31,32,33].

However, traditional harvesting methods historically used to source such ingredients have raised environmental sustainability concerns. Overharvesting can disrupt delicate marine ecosystems, and sourcing ingredients from the wild can lead to inconsistencies in quality and supply. Microalgal cultivation in a controlled, enclosed environment may offer a preferable alternative, eliminating the risk of overharvesting and ensuring consistent supply and quality [30].

The spray zone is the upper, most extreme part of the intertidal domain—regularly splashed, but never covered by the ocean. Life in the spray zone must adapt to exposure to the air, sun, rain, varying temperatures, and varying salinity. *Porphyridium cruentum* (also known as *Porphyridium purpureum* in some taxonomic classifications) is a unicellular red microalga belonging to the family *Porphyridiophyceae*, that has adapted to this spray zone [34,35]. *Porphyridium*’s distinctive red-purple coloration derives from the photosynthesis-related pigment phycoerythrin. Its cells are spherical in shape with diameters ranging from 6 to 10 μm and, critically, lack a rigid cell wall. As part of its adaptation to the spray zone, *Porphyridium* produces a sulfated polysaccharide (up to 100% of its weight), which is excreted outside of the cell membrane to form a mucilaginous protective hydrogel layer (in lieu of the missing cell wall). This hydrogel shell keeps *Porphyridium* moisturized and protected from environmental stressors. As a result, *Porphyridium* has been extensively studied for its ability to produce high-value biomolecules, first and foremost exopolysaccharides (EPSs).

*Porphyridium*’s EPS can comprise up to 57% of the dry weight biomass, and has been characterized as a heteropolymer of molecular weights ranging as widely as 140 kD to 7000 kD, bearing about 7% sulfation, composed mainly of xylose (20–50%), glucose (10–30%), galactose (20–40%), and glucuronic acid (up to 12%) [36,37,38,39,40,41,42,43,44,45,46]. The presence of galacturonic acid has been reported at least once, but without quantitative information [39]. This EPS is thus negatively charged [26]. Structural studies have identified specific building blocks, including 3-O-(α-D-glucopyranosyluronic acid)-L-galactose, as well as various sulfated derivatives such as glucose 6-sulfate, galactose 6-sulfate, and galactose 3-sulfate. The viscosity of the polysaccharide has been reported to be stable over a wide range of pH values (2–9), temperatures (30–120 °C), and salinities [26,47]. Notably, the *Porphyridium* EPS shows stability to heat and to hyaluronidase degradation [26]; and this EPS has been shown to inhibit hyaluronidase, potentially indicating an ability to delay the enzymatic degradation of HA [48].

*Porphyridium cruentum* EPS has been shown to display multiple biological activities relevant to cosmetic and dermatological applications. These include antioxidant, anti-inflammatory, anticancer, and UV protective properties [49,50,51,52,53]. Studies by Ahn et al., *inter alia*, have indicated that EPS from *P. cruentum* can enhance skin health through diverse mechanisms, including improving skin moisturization and barrier function, elasticity, and improved cell migration, promoting wound healing [51]. These effects implicate numerous key skin tissue factors, including aquaporin-3, filaggrin, involucrin, loricrin, elastin, and fibrillin-1. The biological activity of EPS appears to bear some correlation to sulfur content. The alga also contains substantial amounts of protein (up to 39% of biomass dry weight), lipids (including unsaturated fatty acids eicosapentaenoic acid, docosahexaenoic acid, and arachidonic acid), as well as carotenoids such as zeaxanthin [35,54].

Polydeoxyribonucleotides (PDRNs) are bioactive DNA fragments (with molecular weights ranging approximately 50–1500 kDa), known for their anti-inflammatory and regenerative benefits on skin [55,56,57]. Many of the effects of PDRN’s are thought to proceed (via the selective activation of the human A2a adenosine receptor ADORA2A), and include the promotion of wound healing, tissue repair, cell proliferation and migration (including keratinocytes and fibroblasts), angiogenesis (via increased vascular endothelial growth factor expression), and of the synthesis of collagen and HA (via upregulation of HA synthases), and the reduction of inflammation [55,56,57,58,59,60,61,62,63,64,65,66,67]. It is also thought that PDRNs may provide nucleotides through DNA salvage pathways, contributing to their effect on cellular proliferation and regeneration. These multiple, clinically relevant biological properties have led to the application of topically applied PDRNs to skin hydration, inflammation reduction, anti-aging, and skin regeneration, including in combination with HA [55,68].

Traditionally, PDRNs have been extracted primarily from the sperm cells of salmonid fish species, specifically salmon trout (*Oncorhynchus mykiss*) or chum salmon (*Oncorhynchus keta*) [56,69]. Commercial PDRN products contain mixtures of deoxyribonucleotides, predominantly in the 80–200 kDa range with a peak molecular weight at approximately 132 kDa.

Recent research has also explored alternative marine sources. PDRN has been extracted from red macroalga *Porphyra* (also known as nori), and shown to possess anti-inflammatory effects in a macrophage cell model [70]. And very recently, PDRN derived from green microalga *Chlorella protothecoides* has demonstrated efficacy towards enhancing skin regeneration and wound healing through an ADORA2A-dependent mechanism [65]. However, to the best of our knowledge, the presence of PDRNs in *Porphyridium* extracts has not been previously reported.

Based on the recorded properties of these components, and the similarities with HA suggested both by these properties’ end benefits and by the EPS’s structure, we thus aim here to show for the first time the biological activities and topical benefits of a *Porphyridium* extract containing both the characteristic sulfated exopolysaccharides as well as endogenous PDRNs derived from the microalgal culture itself, and thereby demonstrate its potential as an effective alternative to HA in topical cosmetic applications. 

## 2. Results

### 2.1. In Vitro Results

#### 2.1.1. ADORA2A Receptor Activation

The *Porphyridium* extract’s ability to activate the human ADORA2A receptor, key mediator of the biological effects of PDRNs, was evaluated in a CHO cell model expressing this receptor, against a 5′-(*N*-Ethylcarboxamido)adenosine (NECA) control.

**Figure 1:**
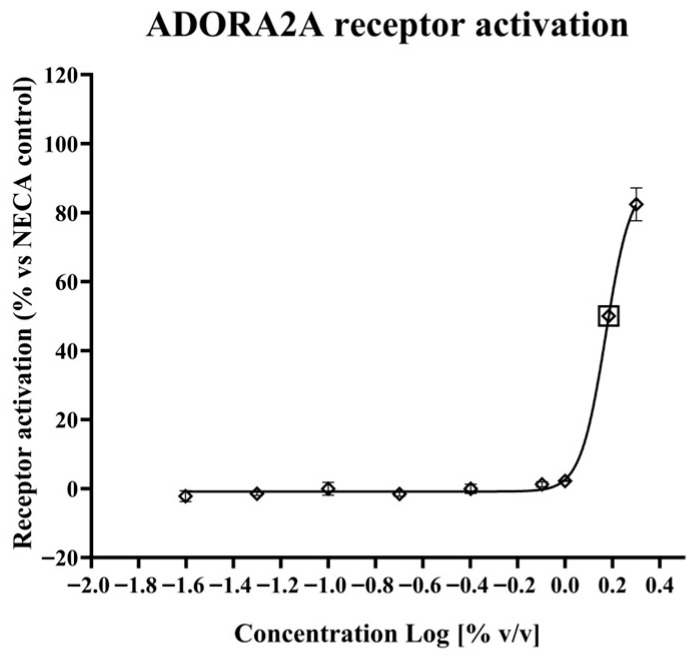
Effect of the *Porphyridium* extract on ADORA2A receptor activation, as % of 6 µM NECA control. Lozenges indicate experimental data points (average of *n* = 3). Error bars indicate standard deviation. The curve represents a nonlinear least-squares fit equation (R^2^ = 0.9963). The calculated EC50 point is shown by a lozenge enclosed in a square.

The resulting data (Figure 1) showed that the *Porphyridium* extract does effectively activate the ADORA2A receptor, with an EC50 of ca. 1.53% extract in medium, and maximum activation of 82.43% vs. the NECA control at the highest concentration tested (2% extract in medium).

#### 2.1.2. Effect on Pro-Collagen I and Collagen III Production

The *Porphyridium* extract’s effect on the production of collagens was evaluated through the quantification of collagen I precursor pro-collagen I (by ELISA) and of collagen III (by immunofluorescent staining) in normal human dermal fibroblasts (NHDF) cultures.

**Figure 2:**
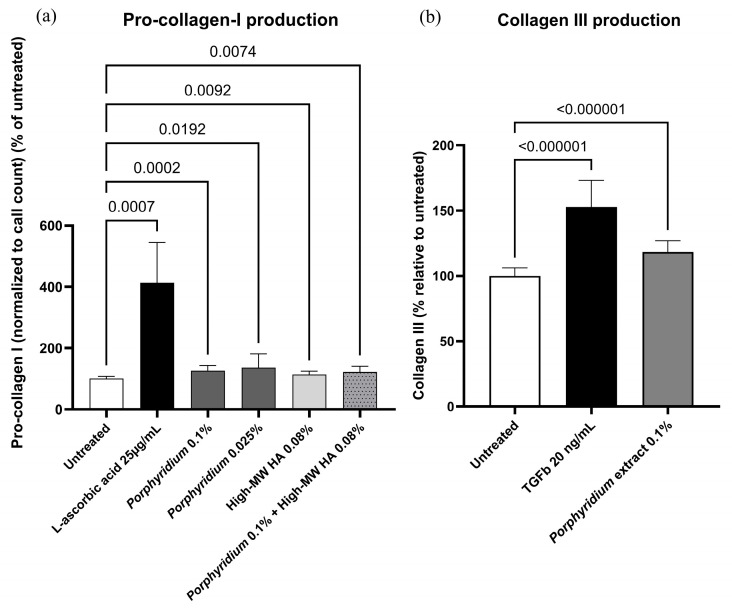
Effect of the *Porphyridium* extract on production of pro-collagen I (**a**) and collagen III (**b**) in NHDF cultures vs. untreated and positive controls. Average of *n* = 8–12 for pro-collagen I, and *n* = 15 for collagen III. Numbers above the bracket indicate the statistical *p*-values for the comparison indicated. MW = molecular weight; HA = hyaluronic acid.

Treatment with 0.025% or 0.10% *Porphyridium* extract resulted in significant increases in NHDF pro-collagen I production (+36%, *p* = 0.0192, at 0.025%), as shown in Figure 2a. For comparison, treatment with 0.08% high-molecular-weight HA led to an increase of only 14% in pro-collagen production (*p* = 0.0092 vs. untreated), and the combination of 0.1% *Porphyridium* extract and 0.08% High-MW HA did not improve the results compared to extract alone (+22% vs. untreated, *p* = 0.0074). The ascorbic acid control (25 µg/mL) produced the expected strong increase in pro-collagen production (+313%, *p* = 0.0007).

Treatment with 0.10% *Porphyridium* extract also resulted in a significant increase in NHDF collagen III production (+18%, *p* < 0.000001), as shown in Figure 2b. The TGFβ control (20 ng/mL) produced the expected increase in collagen III production (+53%, *p* < 0.000001).

#### 2.1.3. Effect on Hyaluronic Acid Production

The *Porphyridium* extract’s effect on the production of HA was evaluated by ELISA in a culture of normal human dermal fibroblasts (NHDF).

**Figure 3:**
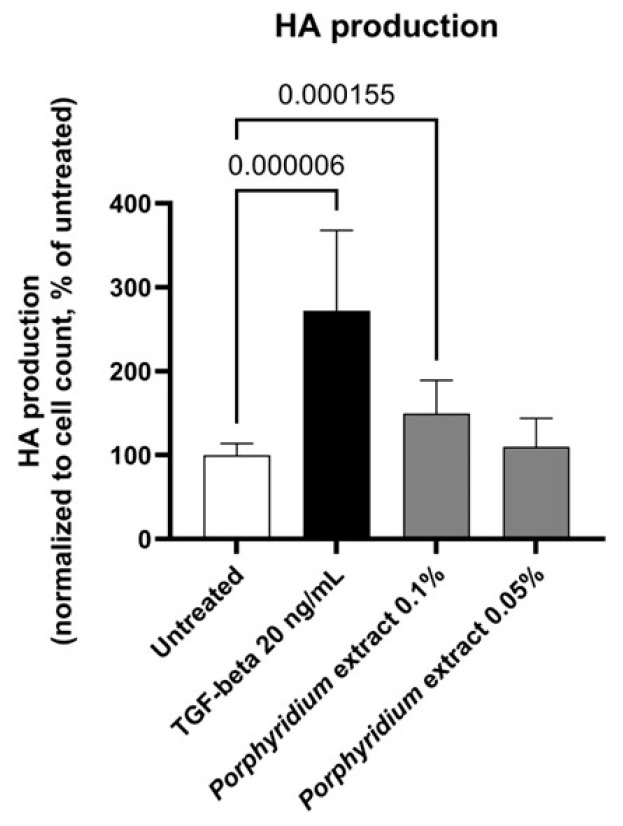
Effect of the *Porphyridium* extract on hyaluronic acid production in an NHDF culture vs. untreated control. Average of *n* = 15–16. Numbers above the bracket indicate the statistical *p*-values for the comparison indicated.

Treatment with *Porphyridium* extract resulted in a significant increase in HA production by NHDF (+50%, *p* = 0.000155, with 0.10% extract), as shown in Figure 3. The TGF-β control (20 ng/mL) produced the expected strong increase in HA production (+172%, *p* = 0.000006).

#### 2.1.4. Anti-Inflammatory Effect

The *Porphyridium* extract’s anti-inflammatory effect was assessed by ELISA measurement of IL8 released in a culture of normal human dermal fibroblasts (NHDF) stimulated by 0.002 ng/mL IL1-α.

**Figure 4:**
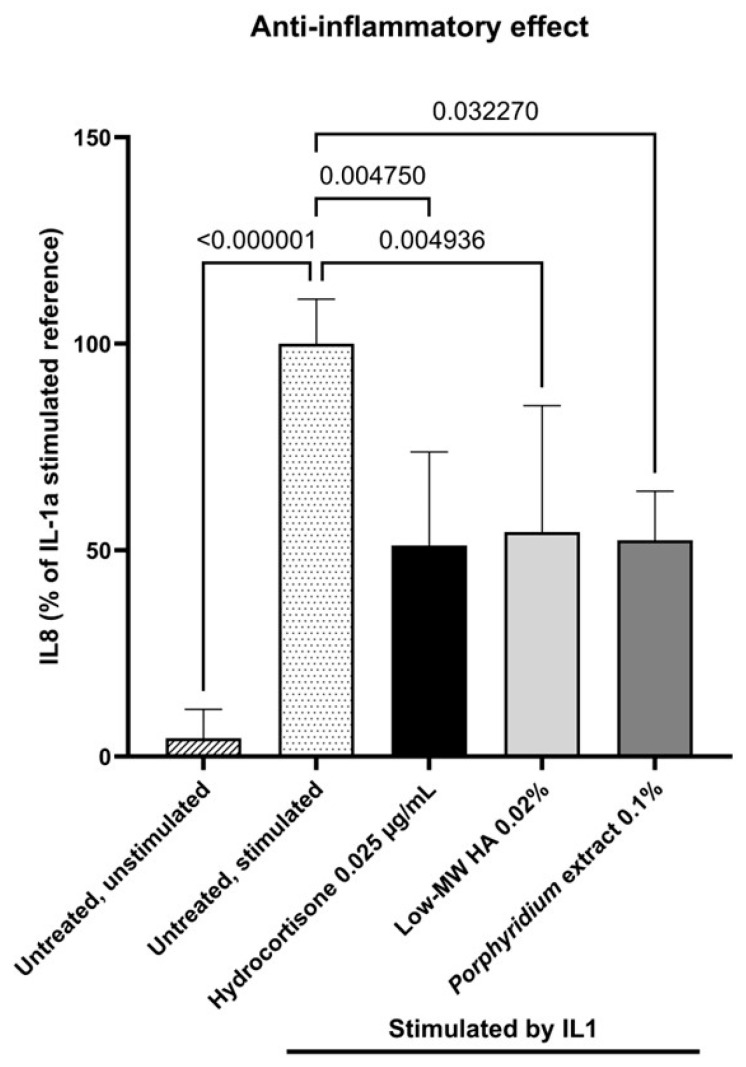
Effect of the *Porphyridium* extract on IL8 production in an NHDF culture stimulated with IL1-α vs. untreated control. Average of *n* = 9–12. Numbers above the bracket indicate the statistical *p*-values for the comparison indicated. MW = molecular weight; HA = hyaluronic acid.

Treatment with 0.10% *Porphyridium* extract resulted in a significant decrease in IL8 production by NHDF (−48%, *p* = 0.032270) compared to the untreated stimulated control, as shown in Figure 4. This effect was comparable to those observed with 0.025 µg/mL hydrocortisone (−49%, *p* = 0.004750, vs. untreated control) and 0.02% low-MW HA (−46%, *p* = 0.004936).

#### 2.1.5. Effect on Autophagy

The effect of the *Porphyridium* extract on autophagic flux was evaluated in a culture of aged normal dermal fibroblasts (sourced from a 67-year-old donor), compared to the autophagic flux in ‘young’ fibroblasts (sourced from a 37-year-old donor) and an untreated aged control.

**Figure 5:**
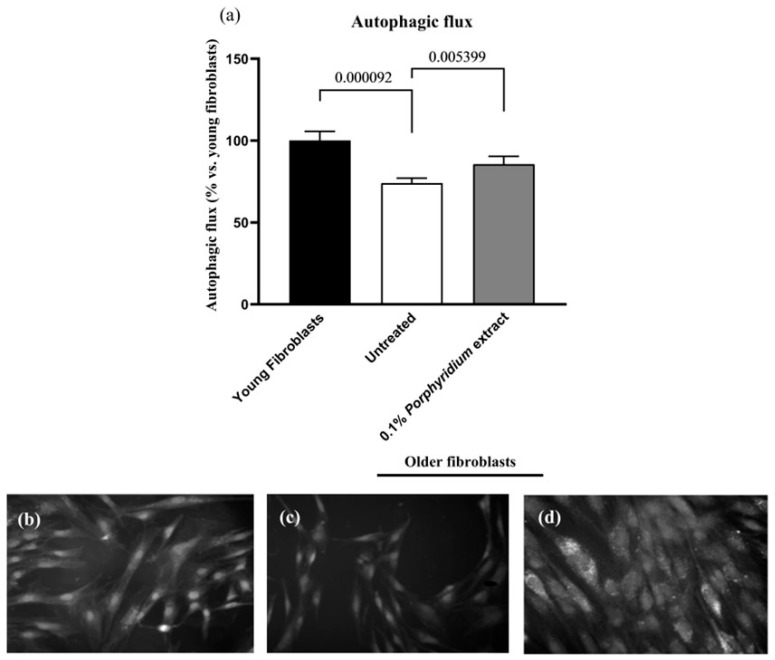
Effect of the *Porphyridium* extract on autophagic flux in an aged NHDF culture vs. aged untreated and young NHDF controls. (**a**) Autophagic flux; (**b**–**d**) illustrative microscopy images: (**b**) young fibroblasts, (**c**) aged fibroblasts, (**d**) aged fibroblasts treated with 0.1% *Porphyridium* extract. Average of *n* = 36. Numbers above the brackets indicate the statistical *p*-values for the comparison indicated.

As expected, untreated older fibroblasts showed a significantly lower autophagic flux vs. younger fibroblasts (−26%, *p* < 0.0001). Treatment with 0.1% *Porphyridium* extract resulted in a noticeable restoration of autophagic flux in the older fibroblasts (+16%, *p* = 0.0054) (Figure 5).

**Figure 6:**
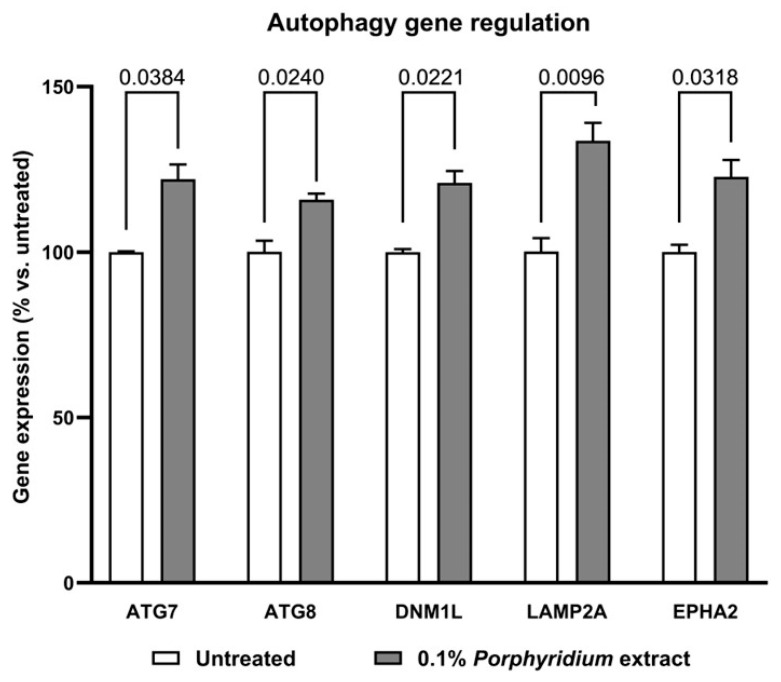
Effect of the *Porphyridium* extract on the expression of autophagy-related genes in an aged NHDF culture vs. untreated control. As analyzed by the ΔΔCt method. Average of *n* = 3. Numbers above the brackets indicate the statistical *p*-values for the comparison indicated. ATG7 = autophagy-related 7; ATG8 = autophagy-related 8; DNM1L = dynamin-1-like; LAMP2A = lysosome-associated membrane protein 2; EPHA2 = ephrin receptor A2.

In an effort to confirm this effect and illuminate the mechanisms behind the *Porphyridium* extract’s pro-autophagic effects, the effect of treatment with the extract on the expression of key autophagy-related genes was evaluated by QPCR in a culture of aged fibroblasts, vs. untreated control (Figure 6).

Significant enhancements were observed in the expression of the following genes, with treatment with 0.1% *Porphyridium* extract compared to the untreated control: ATG7 (Autophagy-related 7), essential for phagophore formation [71,72,73]: +22% (*p* = 0.0384); ATG8 (Autophagy-related 8), essential for the elongation and maturation of autophagosomes [74,75,76,77]: +16% (*p* = 0.0240); DNM1L (Dynamin-1-like), essential for mitophagy [78,79,80]: +21% (*p* = 0.0221); LAMP2A (Lysosome-associated membrane protein 2), essential for chaperone-mediated autophagy [81,82]: +34% (*p* = 0.0096); EPHA2 (EPH receptor A2), essential for lysosome recycling [83]: +23% (*p* = 0.0318).

### 2.2. Clinical Results

Based on the extract’s content of EPS and PDRNs, the EPS’s multicomponent polysaccharide composition, and the preclinical data presented above—including a pro-autophagic effect, an anti-inflammatory effect, and an enhancement in collagen and HA production—this clinical trial aimed at assessing the product’s HA-like benefits in a clinical setting. The treatment’s activity was assessed on parameters typical of HA’s key benefits (including re-plumping, wrinkle reduction, and skin tone/radiance) in a panel composed of volunteers with a history of HA filler use. The active product was compared to a placebo and to a benchmark comprising both low- and high-molecular-weight HA.

#### 2.2.1. Skin Plumpness

**Figure 7:**
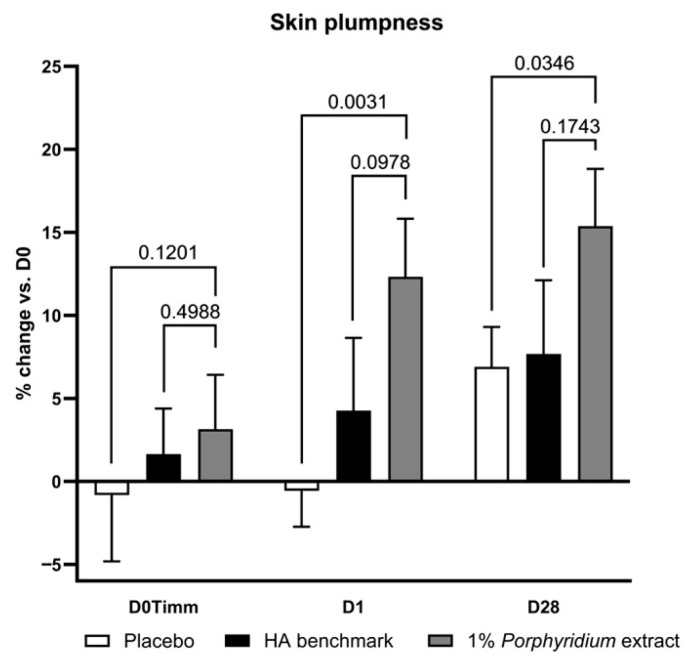
The *Porphyridium* extract delivers improvements in skin plumpness. The effect of topical treatment with the extract on skin plumpness was evaluated by Visia image analysis in a double-blind randomized clinical trial with placebo and HA benchmark controls. Average of *n* = 30–32. Numbers above the brackets indicate the statistical *p*-values for the comparison indicated. D0Timm = 1 h after first application; D1 = 24 h after first application; and D28 = after 28 days’ twice-daily application.

Treatment with 1% *Porphyridium* extract delivered a significant improvement in skin plumpness, as evaluated by image analysis (Visia), after as little as 24 h (after a single application), of 12% vs. D0 and 12% vs. placebo (*p* = 0.0031) (Figure 7). At D28, the improvement vs. D0 reached 15% (+9% vs. placebo, *p* = 0.0346). The active product also shows superior activity vs. the HA benchmark (+8% at both timepoints), albeit this difference does not reach full statistical significance (the effect may be considered borderline, with *p* < 0.10, at D1).

#### 2.2.2. Skin Density

**Figure 8:**
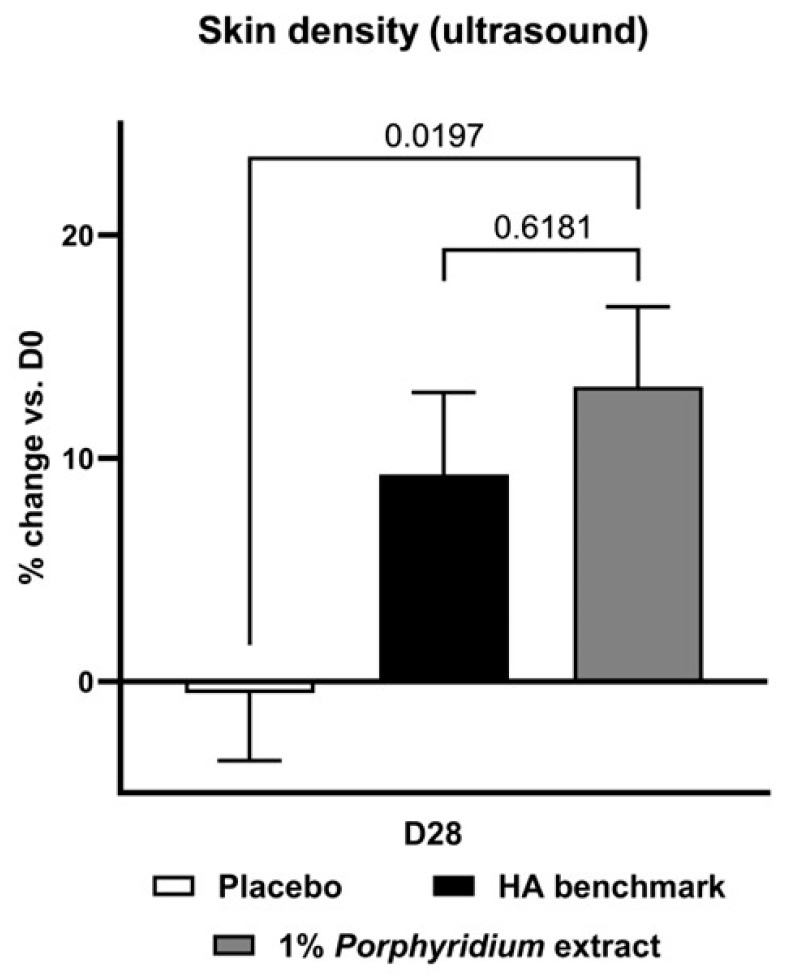
The *Porphyridium* extract delivers improvements in skin density. The effect of topical treatment with the extract on skin density was evaluated by ultrasonography in a double-blind randomized clinical trial with placebo and HA benchmark controls. Average of *n* = 30–32. Numbers above the brackets indicate the statistical *p*-values for the comparison indicated. D28 = after 28 days’ twice-daily application.

At D28, ultrasonography shows that treatment with 1% *Porphyridium* extract strongly increased skin density, with a statistically significant advantage of 14% vs. placebo (*p* = 0.0197) (Figure 8). For its part, the HA benchmark delivered a somewhat lower, though statistically similar, benefit of 10% vs. placebo.

#### 2.2.3. Skin Biomechanics (Elasticity, Firmness)

**Figure 9:**
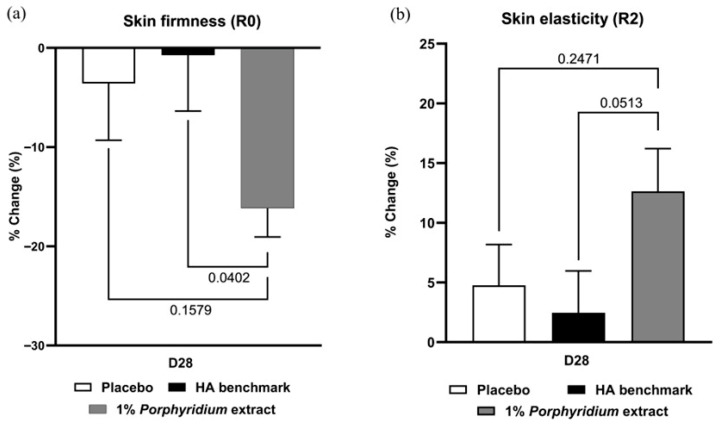
The *Porphyridium* extract delivers improvements in skin biomechanics. The effect of topical treatment with the extract on skin firmness (**a**) and elasticity (**b**) was evaluated by Cutometer in a double-blind randomized clinical trial with placebo and HA benchmark controls. Average of *n* = 30–32. Numbers above the brackets indicate the statistical *p*-values for the comparison indicated. D28 = after 28 days’ twice-daily application.

After 28 days of use, 1% *Porphyridium* extract in formulation delivered a significant improvement in skin biomechanics, as evaluated by Cutometer, showing clear superiority vs. the HA benchmark (+15% Firmness; *p* = 0.0402; and +10% elasticity, *p* = 0.0513) (Figure 9). Note that the HA benchmark formula delivered essentially no benefits in either skin elasticity or firmness at D28 compared to D0, and shows no advantage vs. the placebo formulation (in fact, on both measures, its recorded performance was directionally lower than that of the placebo).

#### 2.2.4. Skin Barrier Function (As TEWL)

**Figure 10:**
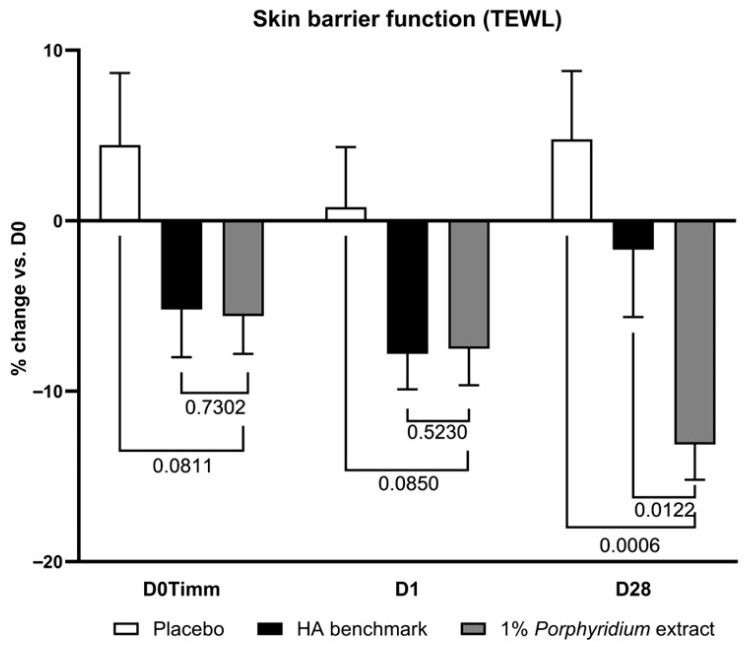
The *Porphyridium* extract delivers improvements in skin barrier function. The effect of topical treatment with the extract on skin barrier function was evaluated by transepidermal water loss (TEWL) in a double-blind randomized clinical trial with placebo and HA benchmark controls. Average of *n* = 30–32. Numbers above the brackets indicate the statistical *p*-values for the comparison indicated. D0Timm = 1 h after first application; D1 = 24 h after first application; and D28 = after 28 days’ twice-daily application.

After 28 days of use, 1% *Porphyridium* extract in formulation delivered a significant improvement in skin barrier function as evaluated by transepidermal water loss (TEWL) measurement, and showed clear superiority vs. the HA benchmark (TEWL −13% vs. D0; −18% vs. placebo, *p* = 0.0006; and −11% vs. benchmark, *p* = 0.0122) (Figure 10). The active product also delivers a benefit after a single application, at 1 h and 24 h (−10, −8% TEWL vs. placebo, although only with borderline statistical significance: *p* = 0.0811 and 0.0850, respectively), matching the benchmark’s performance.

#### 2.2.5. Skin Radiance

**Figure 11:**
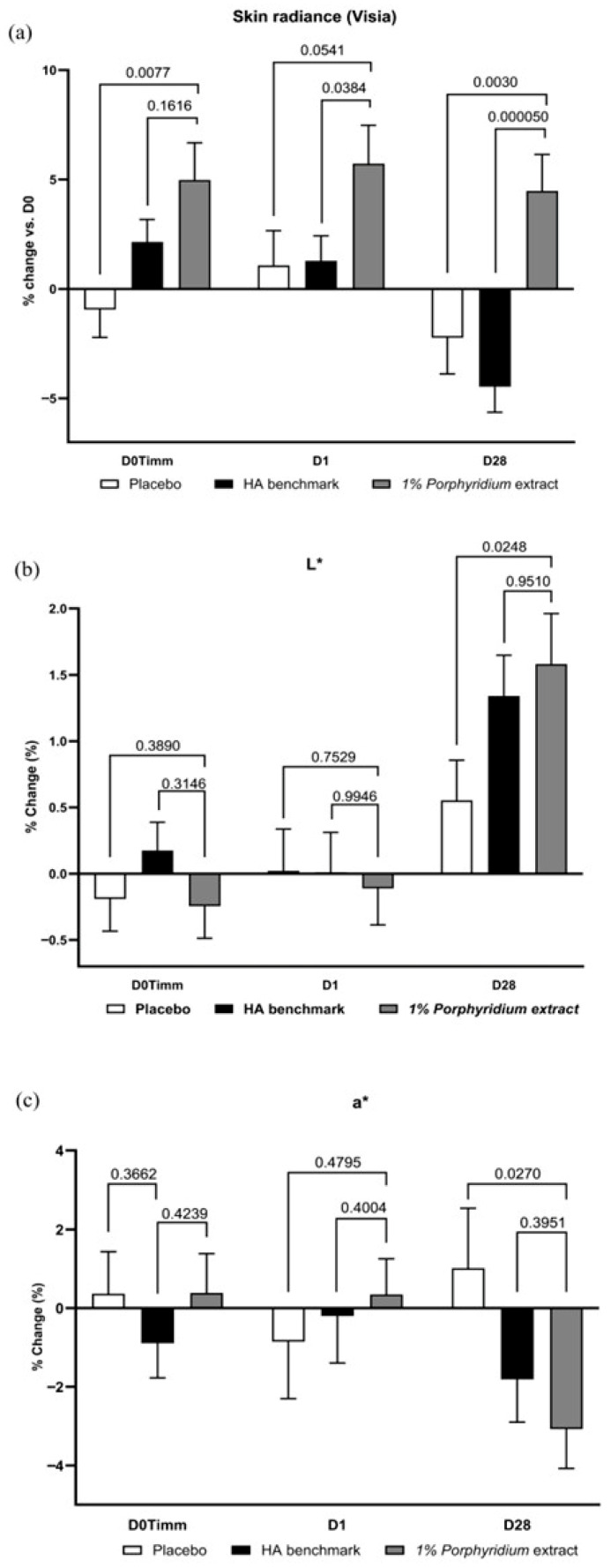
The *Porphyridium* extract delivers improvements in skin radiance and skin tone. The effect of topical treatment with the extract on skin radiance and skin tone was evaluated by Visia image analysis in a double-blind randomized clinical trial with placebo and HA benchmark controls. (**a**) Skin radiance; (**b**) luminance (L*); (**c**) redness (a*). Average of *n* = 30–32. Numbers above the brackets indicate the statistical *p*-values for the comparison indicated. D0Timm = 1 h after first application; D1 = 24 h after first application; and D28 = after 28 days’ twice-daily application.

In Visia image analysis, 1% *Porphyridium* extract in formulation demonstrated a very consistent advantage in enhancing skin radiance at all time points (+6%, *p* = 0.0077 at 1 h; +5% at 24 h, *p* = 0.0541; and +7% at D28, *p* = 0.0030) vs. the placebo formulation, which has little to no effect (Figure 11). The active product also showed a significant advantage vs. the HA benchmark (+4%, *p* = 0.0384 at 24 h and +9%, *p* < 0.0001 at D28). At D28, a *negative* effect was now observable with the benchmark (−4% vs. D0), even though at 1 h the benchmark showed a slight positive effect, with borderline statistical significance vs. placebo.

At D28, image analysis also reveals that the active delivers a significant increase in luminance (L*, +1% vs. placebo, *p* = 0.0248) and a strong decrease in redness (a*, −4% vs. placebo, *p* = 0.0270). These effects are statistically significant vs. placebo, and tend to outperform the effect of the HA benchmark, albeit this difference is not statistically significant. 

#### 2.2.6. Wrinkle Reduction

**Figure 12:**
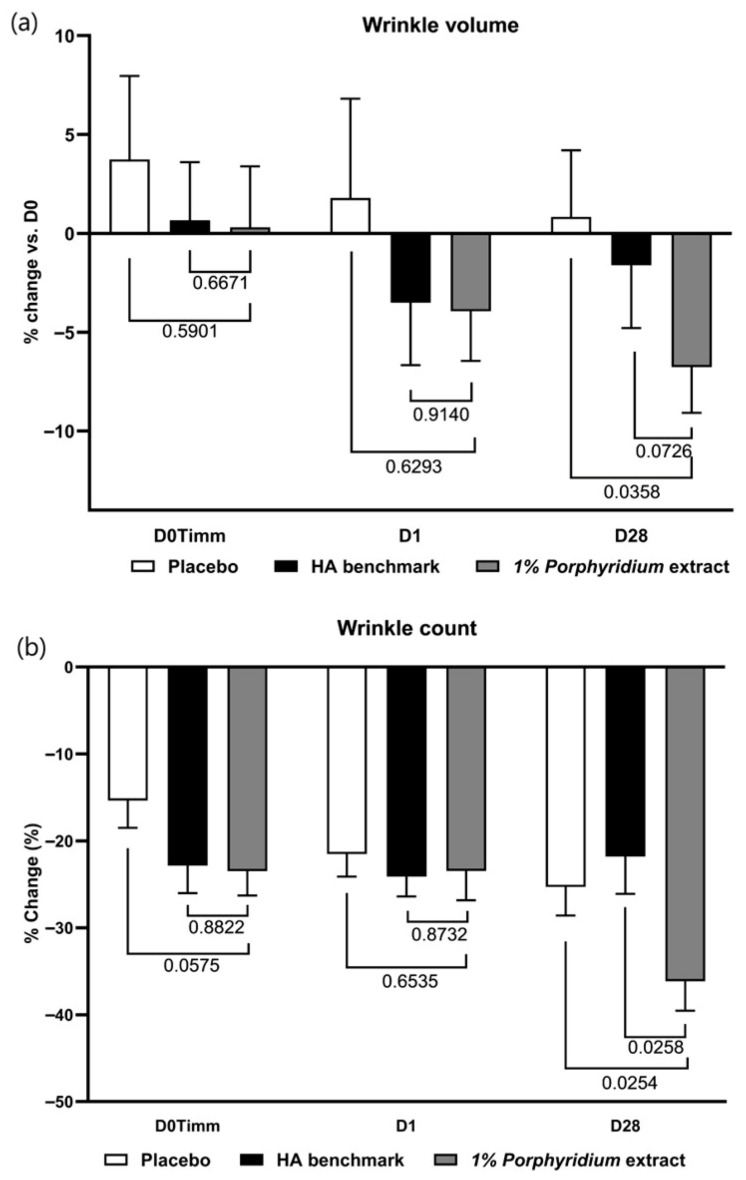
The *Porphyridium* extract delivers improvements in skin wrinkling. The effect of topical treatment with the extract on wrinkle volume (**a**) and wrinkle counts (**b**) was evaluated by Visia-PRIMOS image analysis in a double-blind randomized clinical trial with placebo and HA benchmark controls. Average of *n* = 30–32. Numbers above the brackets indicate the statistical *p*-values for the comparison indicated. D0Timm = 1 h after first application; D1 = 24 h after first application; and D28 = after 28 days’ twice-daily application.

After 28 days of use, 1% *Porphyridium* extract delivers a significant anti-wrinkle effect, with a statistically significant advantage vs. placebo of 8% (*p* = 0.0358) in Visia-PRIMOS image analysis of wrinkle volume (Figure 12) (and also showing a 5% advantage in wrinkle volume vs. the HA benchmark, albeit with only borderline statistical significance, *p* = 0.0726). An effect of similar magnitude is observed in clinician grading at D28. At 1 h and 24 h, generally similar trends are observable, at lower magnitudes.

Wrinkle count data shows a statistically significant advantage for the active vs. both the placebo and the HA benchmark (respectively −11%, *p* = 0.0254 and −14%, *p* = 0.0258) at D28. In this measure, the HA benchmark showed no advantage vs. placebo.

#### 2.2.7. Skin Roughness

**Figure 13:**
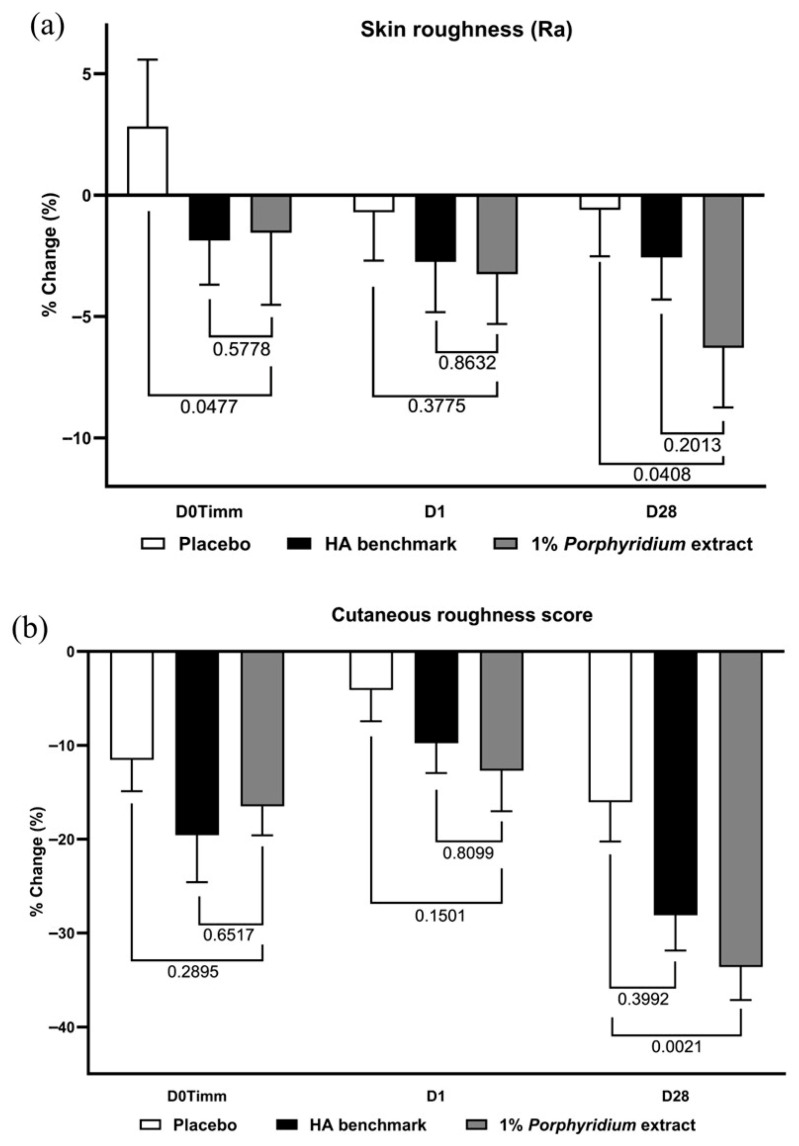
The *Porphyridium* extract delivers improvements in skin roughness. The effect of topical treatment with the extract on skin roughness was evaluated by Visia-PRIMOS image analysis and clinician grading in a double-blind randomized clinical trial with placebo and HA benchmark controls. Average of *n* = 30–32. Numbers above the brackets indicate the statistical *p*-values for the comparison indicated. (**a**) Visia-PRIMOS image analysis (Ra index); (**b**) clinician grading.

1% *Porphyridium* extract in formulation exerted a significant skin smoothing effect after 28 days of use, observed both in instrumental data (−6% Ra vs. placebo, *p* = 0.0408) and even more clearly in clinician grading (−18% vs. placebo, *p* = 0.0021) (Figure 13). In both cases, the active product tended to outperform the HA benchmark, albeit without statistical significance between the two. At 24 h, the same effects are already observable, although at a lower magnitude and without statistical significance. At 1 h (D0Timm), the HA benchmark shows a smaller but statistically significant effect on Ra vs. the placebo, and the active tends to match the benchmark’s performance. 

#### 2.2.8. Spot Reduction

**Figure 14:**
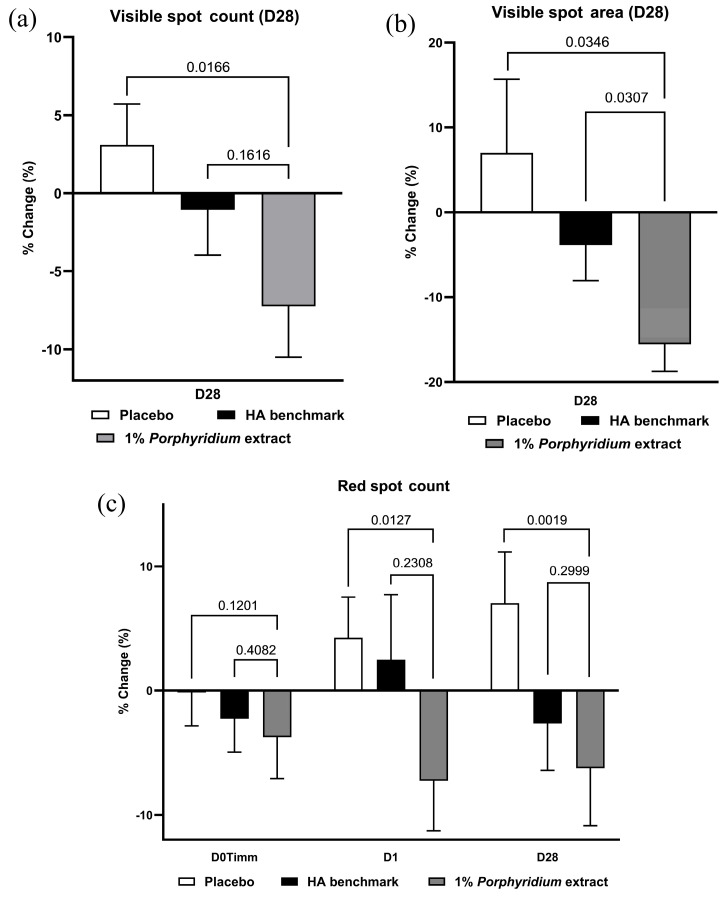
The *Porphyridium* extract delivers improvements in skin spots. The effect of topical treatment with the extract on skin spot counts was evaluated by Visia image analysis in a double-blind randomized clinical trial with placebo and HA benchmark controls. Average of *n* = 30–32. Numbers above the brackets indicate the statistical *p*-values for the comparison indicated. (**a**) Visible spot counts; (**b**) visible spot areas; (**c**) red spot counts.

After 28 days of use, 1% *Porphyridium* extract in formulation delivered a strong and statistically significant reduction in visible spot counts (−10% vs. placebo, *p* = 0.0166) and area (−23% vs. placebo, *p* = 0.0346). The latter also shows a statistically significant advantage (−12%, *p* = 0.0307) vs. the HA benchmark. (Figure 14a,b). 

Data on brown and UV spots shows significant trends: brown spot areas show statistically significant advantages for the active product, of −12% and −9% vs. placebo and HA benchmark, respectively, with brown spot counts showing a statistically significant advantage vs. placebo. UV spot counts show statistically significant advantages, also of −12% and −9% vs. placebo and HA benchmark, respectively.

Finally, red spot counts data show a significant advantage at D28 vs. the placebo (−13%, *p* = 0.0019) and HA benchmark (−4%, without statistical significance) (Figure 14c). At 24 h, a similar advantage is observable (−12% vs. placebo, *p* = 0.0127). It may be noted that the effect of the placebo formulation appears to worsen over time. Red spot area data shows similar trends.

## 3. Discussion

We report herein on the biological activities of an EPS- and PDRN-rich *Porphyridium cruentum* extract. While the EPS has been widely described previously, as discussed above, we believe that the presence of PDRNs in *Porphyridium* extracts has not been previously reported. Several explanations for the presence of PDRNs in the extracellular medium from which this extract is produced are possible. These PDRNs may have been excreted outside of living cells (e.g., as cargo in extracellular vesicles). Indeed, many microorganisms naturally release DNA into their extracellular environment, as part of normal metabolic processes or in response to environmental conditions [84,85]. This can serve various functions, such as a stress response, intercellular communication, or genetic exchange. In high-density *Porphyridium* cultures, DNA accumulation in the extracellular polysaccharide matrix could account for part of the extract’s PDRN content. The presence of PDRNs in the extract could also be the result of cell breakage, releasing intracellular contents including fragmented DNA, either during cultivation (as a proportion of cells may be expected to die of natural causes, especially during the stationary phase where cell population reaches an equilibrium), or during harvesting, especially due to the mechanical stresses involved in the separation of biomass by centrifugation. In whichever manner they are released, these DNA fragments may integrate into the EPS matrix, where they could then be expected to be retained through the subsequent ultrafiltration step and carried over into the final extract. The small size of the PDRNs observed in the extract suggests that an enzymatic process is involved (before or after release from the *Porphyridium* cell), rather than purely mechanical phenomena.

The enhanced pro-collagen I and collagen III synthesis we observed in vitro with the *Porphyridium* extract is reminiscent of what has been reported for at least some HA molecular weight ranges, suggesting that the extract may indeed possess at least some HA-like biological properties, as was suggested by the structural similarities between HA and *Porphyridium*’s EPS (similarities including monosaccharide makeup, electric charge, and molecular weight distributions). The effect is also consistent with the presence of PDRNs in the extract. The enhanced HA synthesis we observed in a similar cell culture also conforms to known activities of PDRNs, as noted above, and thus may possibly be due to their presence in the extract. The extract’s anti-inflammatory activity, demonstrated through the lowering of IL8 under IL1-α stimulation, further reinforces both indications.

The extract’s pro-autophagic effect may also be qualified as HA-like, based on a parallel with the enhancement of autophagy by HA, as noted above. In the *Porphyridium* extract’s case, while the increase in autophagic flux observed in older fibroblasts with the extract did not fully restore the autophagic flux to its level in the younger cells, it did close approximately half of the gap measured between untreated younger and older cells. Considering the 30-year difference between the donors used, this effect might be described as having rejuvenated the older cells’ autophagic processes by approximately 18 years, a non-trivial number. The results of gene expression evaluation backed and elucidated this effect on the mechanistic level by showing increased expression of key autophagic genes; each significant in its own right but all the more notable given that taken together, this set of genes essentially covers every critical stage along the whole autophagy chain, from start to finish: ATG7 (autophagy-related 7), which starts the autophagic process by helping form autophagosomes, the structures that engulf and recycle cellular waste [71,72,73]; ATG8 (autophagy-related 8), which is also key for building and organizing autophagosomes, attaching to the autophagosome membrane and helping recognize and recruit cargo for recycling (particularly its family member LC3) [74,75,76,77]; DNM1L (dynamin-1-like), which assists mitophagy in particular, and also helps recruiting autophagy machinery to damaged mitochondria [78,79,80]; LAMP2A (lysosome-associated membrane protein 2), which is an important gatekeeper in chaperone-mediated autophagy, ushering proteins into lysosomes for degradation [81,82]; and EPHA2 (ephrin receptor A2), which plays a complex role in the final stages of recycling in autophagy, and in autophagy regulation [83]. Conversely, it is both notable and interesting that in this case, the extract’s properties differ from those reported for PDRNs alone, which have recently been shown to inhibit autophagy [86]. This may indicate that the extract’s pro-autophagy effects are not linked to the PDRNs it contains, but rather to some other component (possibly, the EPS), whose effect more than overcomes any anti-autophagic effect of the PDRN; seen in this light, the extract’s composition appears to offer a notable best-of-both-worlds combination of HA-like and PDRN-like effects, without many of the drawbacks of either single benchmark component.

Our clinical data further backs this assessment with a clear demonstration of the benefits of the extract in real-life use, as evaluated against a placebo and an HA benchmark containing both high- and low-molecular-weight HA fractions. These benefits were shown to include significant improvements in skin plumpness and skin density, skin elasticity and skin firmness, skin barrier function, skin radiance, luminance, redness, wrinkling, roughness, and spot reduction (visible, brown, UV, and red). Some of these effects were rapid, observable after a single product application (e.g., on skin plumpness, skin barrier function, wrinkling, roughness, and red spots), suggesting either physical mechanisms or rapid biological effects. It may be suggested that at least some of the effects observed, namely those linked to abnormal or excessive pigmentation and/or damaged tissue—e.g., visible, brown, and UV spots, as well as the overall skin lightening effect recorded as an increase in luminance and which also contributes to the increase in skin radiance—may be attributable to the pro-autophagic properties of the extract, in that this activity may lead to increased degradation of excess melanin, and thus to a reduction in hyperpigmentation. This is supported by research from Murase et al. and from Kim et al., who demonstrated the role of autophagy in regulating pigmentation through melanosome degradation [87,88]; and conversely, by parallel research indicating that deficiencies in autophagy can lead to hyperpigmentation phenomena [89,90]. The same pro-autophagic effect may also be thought to contribute (together with the enhancement in matrix protein production discussed above) to the enhancement in skin biomechanical properties (elasticity, firmness), through the increased removal of damaged and malfunctioning matrix protein; this is supported by research from Tashiro et al., among others [91,92]. Conversely, the HA benchmark exhibits some notable time-related behavior over the course of the study. In some parameters (skin barrier function, skin radiance), initial benefits recorded after the first application are no longer observable at D28. This may hint that competing mechanisms are at play with HA. For example, we may be observing an instant benefit from physical (skin barrier) or optical (radiance) effects of the higher-molecular-weight HA fractions depositing at the skin surface; and then seeing this effect eventually overcome by irritating (or even pro-inflammatory) effects of HA, especially of its lower-molecular-weight fractions [10,93,94]. Notably, although the *Porphyridium* extract did not significantly exceed the performance of the HA benchmark in all measured parameters, it consistently matched that benchmark on most measured parameters (including wrinkling and roughness parameters, luminance, redness, skin density, and plumpness); and further, exhibited clear, statistically significant superiority vs. the HA benchmark in several key parameters (e.g., skin elasticity and skin firmness, on which the HA benchmark appeared to deliver essentially no benefit; skin barrier function; wrinkle counts; skin radiance; and visible and red spot reduction). Considering the clinical results as a whole, it may be said that the *Porphyridium* extract consistently and reliably either matched or exceeded the effects of the HA benchmark—thus confirming its HA-like properties, and suggesting significant advantages in selected performance parameters.

Significant areas remain where improvements may be suggested to the work presented here. For one, it would be very interesting to delve further into the structure of the *Porphyridium* polysaccharide, seeking a better understanding of the different fractions present, their saccharide makeup, molecular masses, volumes, and three-dimensional structures—especially, if concurrently, one could establish correlations with some of the observed biological activities of the material, and perhaps shed light on possible synergistic effects between the different fractions. NMR data might yield interesting additional insights, for example. Furthermore, it might be valuable to back these observations with examinations of the skin penetration behavior of these same fractions, seeking to confirm their assumed differential depth penetration and further confirming the attribution of various effects to specific fractions.

Our observations on the presence of PDRNs in the extract also suggest additional possibilities. It might be interesting to investigate the specific origin of these PDRN fragments (organic release pre-harvest or mechanical release during harvest; role of enzymatic processes pre- or post-release from the cell, etc.), and the use of cultivation and process parameters to control the quantity and quality (e.g., size distribution) of PDRN in the extract.

There also likely remain valuable additions to be made to the in-vitro data set presented above. As a beginning, the studies presented herein may be strengthened by repetition using a higher number of donors, covering a broader range of ages (and especially, higher age ranges), perhaps as well as both sexes and a variety of ethnic backgrounds.

The link, suggested here, between the ADORA2A activation shown and the clinical effects observed benefits, is based on the literature discussed above, as well as on the preclinical data presented, which supports an interpretation of the data both as showing downstream effects of ADORA2A activation and as providing mechanistic elucidation for the clinical benefits. Nevertheless, it should be acknowledged that this does not constitute an unequivocal causal demonstration of said link. Additional studies, for example, ones including ADORA2A receptor blocker/antagonist controls, may be useful in that regard.

Likewise, while our data on the effects of the *Porphyridium* extract on autophagy, and the related literature discussed above, argue for a role for autophagy in at least some of the clinical effects we observed, it should be acknowledged that these also do not unequivocally establish a causal involvement of autophagic flux. For this purpose, additional studies incorporating autophagy/lysosomal inhibitor controls might be considered to provide additional confirmation of the mechanisms suggested here.

More broadly, it might be interesting to examine additional known endpoints of HA and/or PDRN activity in simple cell culture models, including other ECM proteins or effects on cell proliferation (keratinocytes, for instance) and wound healing. Valuable further insights may also be gleaned from testing the extract in more elaborate models, starting with *ex-vivo* tissue models; these may include classical explant models, or models highlighting specific aspects of skin biology, e.g., including model microbiomes (to examine any possible role of the extract’s reported antimicrobial properties), or including some immune capability (to further probe into the extract’s anti-inflammatory, or at least non-inflammatory, effects as compared to low-molecular-weight HA fractions). Studying the effects of the *Porphyridium* extract in some three-dimensional wound or disease models (e.g., atopic dermatitis, psoriasis, or diabetic wound models) may also yield valuable data.

The clinical research described herein might also be expanded, in particular with a significantly longer study (with a duration measured in months instead of weeks), to more fully and reliably assess the effects of the extract over the long term, and especially its anti-aging effects. Similarly, additional HA-like benefits of relevance to the cosmetic industry might be profitably examined in additional clinical trials—including for example, the effects of the extract on lips, the eye contour area, or in hand and body skin care.

In conclusion, we have presented here a microalgal exopolysaccharide- and natural PDRN-rich extract from *Porphyridium cruentum*, whose structural and biological similarities with hyaluronic acid suggested the possibility of hyaluronic-like properties and benefits. These similarities included polysaccharide composition, electrical charge, molecular weight distribution, and the known downstream effects of PDRNs and adenosine receptor activation.

As anticipated, we demonstrated that like HA and/or PDRNs, this extract increases collagen and HA synthesis, reduces inflammation, restores autophagy in aged fibroblasts, strengthens skin barrier function, increases skin plumpness and density, and reduces the appearance of spots and wrinkles—resulting in a more radiant, youthful, and plumped skin appearance. It may be suggested that, similarly to what is known of HA, some of these effects may be attributable to the polysaccharide’s high-molecular-weight fractions acting on the skin surface, and other, smaller ones (possibly, together with the extract’s PDRNs) penetrating deeper into the tissue to modulate key biological functions there.

Notably, in our clinical trial, a formulation containing 1% *Porphyridium* extract gel consistently matched or, on key selected parameters, significantly exceeded the performance of a relevant benchmark product containing a total of 1% HA, made up of both high- and low-molecular-weight grades.

Taken together with additional advantages of this extract, not least the EPS’s resistance to degradation by hyaluronidase, and including its non-animal origin and the highly scalable, sustainable, and controllable nature of its production process, we believe that these results demonstrate that this extract is a natural, cost-effective for industrial cosmetic manufacturing, safe, and highly efficacious alternative to hyaluronic acid in topical cosmetic formulations, consistently matching the HA benchmark’s performance and exceeding it on several measured parameters.

## 4. Materials and Methods

### 4.1. Material—Porphyridium Cruentum Extract

Using a proprietary process modified from technology reported by Arad and others [26,95,96,97,98,99,100], *Porphyridium cruentum* was cultivated in artificial seawater, in outdoor vertical reactors illuminated by natural sunlight (Israel), while maintaining pH at 7.0–8.0 via introduction of carbon dioxide (CO_2_), and culture temperature at 22–26 °C. The culture was harvested once reaching a density of 20–50 × 10^6^ cells/mL and a viscosity of 10–50 cp. After centrifugal separation of the biomass itself, the medium was concentrated by ultrafiltration (0.002-µm cutoff). For in-vivo testing, the extract was sterilized, and preserved with 0.4% sodium benzoate (Lanxess 62609602, Rotterdam, The Netherlands) and 0.4% potassium sorbate (Cooper 1607805, Melun, France), and its pH was adjusted using citric acid (CitriBel 51N, Tienen, Belgium; QS to pH 5.0–6.0, generally in the range of 0.03–0.10%). The final extract’s Brookfield viscosity ranged from 2000 to 8000 cPs.

The resulting EPS showed a wide molecular mass range, from 140 to 6000 kD (as evaluated by size exclusion chromatography: Shodex SB804/806 columns, Munchen, Germany; eluent 0.1 M NaNO_3_ + 0.1 g/L NaN_3_, 0.8 mL/min; static light scattering MALS 18 angle detector; MicroDAWN Wyatt, Santa Barbara, CA, USA); a Brookfield viscosity of no less than 2000 cPs; sulfation of 6–8% (evaluated using the colorimetric assay described by Jaques et al. [101]); and a monosaccharide makeup including 25–40% xylose, 30–40% galactose, 15–25% glucose, 4–8% galacturonic acid, and 5–10% glucuronic acid, as determined by high-performance anion exchange chromatography with pulsed amperometric detection (HPAEC-PAD: DIONEX ICS6000 system, Sunnyvale, CA, USA; equipped with a Dionex CarboPac PA1 250 × 2 mm column thermostated at 28 °C; elution at 0.18 mL/min following a gradient from 0.016 M NaOH to 0.2 M NaOH + 0.5 M sodium acetate) after hydrolysis in 4 N trifluoroacetic acid, for 4 h at 110 °C.

The presence of DNA fragments (PDRNs) was shown by agarose gel electrophoresis (2% agarose gel in TBE buffer; with 6X MassRuler DNA Loading Dye, Thermo Fisher, Waltham, MA, USA; and fluorescent nucleic acid dye GelRed, Merck Millipore SCT123, Waltham, MA, USA). *Porphyridium* extract was loaded at 10% dilution (20 µL), alongside 2.5 and 5 µL of a 25 µg/mL salmon sperm PDRN reference (Invitrogen 15632011, Waltham, MA, USA) and 2.5 μL of each of two size marker ladders (Thermo Fisher SM0383 and SM1213) to allow for an estimation of the size of the DNA fragments. The samples were then migrated under 70 V for 150 min in the TBE buffer. After draining, the final gel was placed into a ChemiDoc MP gel reader (BioRad, Hercules, CA, USA), with detection set at 590 nm. Band intensity was evaluated using the Image Lab 6.1 software (BioRad). The results indicate the presence of a diffuse PDRN band, with sizes falling mostly in the 20–50 bp range (and a minor proportion tailing up to the 200 bp). Quantification indicated a ca. 3–10 ppm DNA fragment content in the extract. This was confirmed by quantifying double-stranded DNA in solution, using a specific, sensitive fluorescent nucleic acid staining method (Quant-iT PicoGreen dsDNA kit, Thermo Fisher P11496). A DNA ladder (Invitrogen SM1213) was used as the reference for quantification. Extract samples were diluted to 2% (*v*/*v*), and the DNA ladder was solubilized at 2000 ng/mL in Tris-EDTA buffer. Briefly, 100 μL of each stock solution was transferred to a 96-half-well black plate, and serial dilutions of the DNA ladder in buffer were performed. Next, 100 μL of PicoGreen dsDNA was added to each well, and the plate was incubated for 10 min with shaking. Following the supplier’s indications, the fluorescence of each well was measured using a Cytation1 Biotek plate reader (Agilent, Santa Clara, CA, USA) (excitation: 480 nm; emission: 520 nm). A calibration curve was calculated based on the DNA ladder readings (yielding an R^2^ value of 1.00). LOD was estimated at 2.0 ng/mL, and LOQ at 7.8 ng/mL. Nine independent batches of *Porphyridium* extract were analyzed, twice each in duplicate (4 measurements total). Once again, the results indicated that the extract samples contained between 3 and 10 ppm DNA fragments, averaging 5.95 ppm with a standard deviation of ±2.49 ppm.

The absence of critical contaminants was ascertained, including: heavy metals (Sb, As, Cd, Cu, Ba, Se, Zn, Cr, Co, Hg, Ni, and Pb; by ICP/MS); allergens (as defined in Annex III of Regulation (EC) n°1223/2009 [102]; by GC-MS); and pesticides (by GC-MC and LC-MS). A microbial load of <10 CFU/g was ascertained (per USP<61>).

Finally, in order to ascertain its safety in human topical use, the extract was submitted to a range of safety and toxicity tests, following gold-standard practices in the cosmetics industry. This included an in vitro model of oral toxicity (3T3 NRU model per OECD 129; in this model, the extract’s estimated LD50 was found to be above 2000 mg/kg); an in vitro model of ocular irritation (human corneal epithelial model per OECD 492; in this model, the extract was classed as non-irritant); an in vitro model of cutaneous irritation (reconstituted human epidermis model per OCED 439; in this model, the extract was classed as non-irritant); an assessment of mutagenic potential (Ames bacterial reverse mutation test per European Directive 2004/10/EC; in this assay, the extract showed no mutagenic or pro-mutagenic activity); an evaluation of potential phototoxicity (UV–visible absorption per OECD 101; this analysis showed no UV absorption in the 2940–400 nm range, and therefore no phototoxic potential is expected from the extract); a ToxTracker genotoxocity study (Toxys BV, Leiden, The Netherlands; in this assay, based on several mouse embryonic stem (mES) reporter cell lines, the extract induced no cellular stress, DNA damage, oxidative stress or unfolded protein response, was therefore assessed to present no risk of mutagenicity or clastogenicity, and was classed as non-genotoxic); and several in-vitro skin sensitization models (Sens-IS, Immunosearch, Grasse, France, in which the extract was classed as non-irritant and non-sensitizing; Keratinosens™, IDEA Lab, Martillac, France, per OECD 442D, in which the extract was classed as non-sensitizing; and a Direct peptide reactivity assay (DPRA) per OECD 442E, in which the extract was again classed as non-sensitizing). The extract was also found to be easily biodegradable after 28 days (OECD 301D), and non-ecotoxic (OECD 201, OECD 202).

Formulability and formulation stability trials were conducted on a simple clear polyacrylate gel formulation and a model oil-in-water emulsion formulation (each containing 2% *Porphyridium* extract), which were shown to be stable based on assessments of pH, Brookfield viscosity, and visual aspect for 1 month at 50 °C, 3 months at room temperature, 4–8 °C, and 45 °C, as well as photostable (vs. UV irradiation) and resistant to centrifugation (3000 rpm, 20 min) and vibration (vibrating table, 400 rpm, 4 h). See Appendix A for full formulation information.

### 4.2. Testing Methods—In Vitro

#### 4.2.1. ADORA2A Receptor Activation

The *Porphyridium* extract’s ability to activate the ADORA2A receptor was assessed in a culture of CHO mitoAeq/Gα16/ADORA2A cells (Axxam, Milan, Italy) stably expressing the human A2a adenosine receptor, alongside a mock cell control (CHO mitoAeq/Gα16). 5000 cells/well were seeded in 384-well plates and incubated overnight at 37 °C, 5% CO_2_ in complete medium (25 µL/well) composed of DMEM-F12 with L-Glutamine and 25 mM Hepes (Euroclone ECM0095L, Pero, Italy), 10% FBS (Merck KGaA 7524, Darmstadt, Germany), 1% Penicillin/Streptomicin (Euroclone ECB3001D), 1% Glutamine Stable (Euroclone ECB3004D), 1 mM sodium pyruvate (Euroclone ECM0542D), 13.5 mM sodium bicarbonate (Euroclone ECM0980D), 5 mg/mL puromycin (InvivoGen ANT-PR-1, San Diego, CA, USA), and 1 mg/mL G 418 Disulfate Salt (Merck G8168) (for ADORA 2A cells only). The culture medium was removed, and 20 µL/well of 0.5X Fluo8 NW calcium-sensitive dye diluted in assay buffer was added (Screen Quest™ Fluo-8 No Wash Calcium Assay Kit, AAT Bioquest 36316, Pleasanton, CA, USA), followed by 1 h incubation at 37 °C.

*Porphyridium* extract at concentrations of 2%, 1%, 0.8%, 0.4%, 0.2%, 0.1%, 0.05% and 0.025% in triplicate or controls NECA (Merck E2387) at 0.00037, 0.0015, 0.0059, 0.023, 0.094, 0.38, 1.50, and 6.00 µM or Adenosine 5′-triphosphate (ATP, Merck A6419) at 0.01, 0.03, 0.10, 0.32, 1.00, 3.16, 10.0, and 31.6 µM in assay buffer, in duplicate, were introduced using the FLIPR^PENTA^ Fluorometric Imaging Plate Reader (Molecular Devices Corp, San Jose, CA, USA). The kinetic response was monitored over 3 min.

Genedata Screener^®^ 21.0.7 (Genedata, Basel, Switzerland) was used for data analysis. The following signal descriptors were calculated from the kinetic data: Baseline (F0) = mean over the period 0–4 s, for evaluation of the fluorescent signal before sample addition; Max on raw trace (Fmax) = max signal over the period 9 s–end; ΔF/F0 = (Fmax − F0)/F0. ΔF/F0 was normalized versus either: stimulator controls (max) and neutral controls (min), to evaluate Activity% versus 6 µM NECA (for Adora 2a cells only); or blank controls (max) and background controls (min) to obtain Activity% versus 31.6 µM ATP (for both cell lines, to enable data comparison between Adora2a and MOCK cells). Dose-response curve fitting was performed using the Analyzer module in Screener^®^ on Activity% values, using the “Smart Fit” option.

#### 4.2.2. Hyaluronic Acid Production in Dermal Fibroblast Culture

NHDF isolated from human dermis (20-, 30- and 35-year-old female donors undergoing plastic surgery and having given informed consent; ethical committee reviews were not required by local regulations (France) for this type of tissue sourcing) were maintained in a specific medium composed of Dulbecco’s Modified Eagle Medium (DMEM) (Dutscher L0060-500, Bernolsheim, France) containing 10% serum (Sigma F7524, Burlington, MA, USA) and 1% antibiotics (penicillin/streptomycin, Sigma P0781) at 37 °C under 5% CO_2_ and 95% humidity. 25,000 cells/well were seeded in 96-well microplates in complete DMEM for 24 h. The culture medium was then replaced with serum-free DMEM for an additional 24 h to promote a quiescent cell state. Then, 10 ng/mL TGF-β or *Porphyridium* extract at 0.05% or 0.10% were then added for an additional 24 h, alongside an untreated control. At the end of the incubation period, supernatants were collected for ELISA (Bio-Techne R&D Systems DY3614, Minneapolis, MN, USA). Briefly, 60 μL of a mixture of 1 μg/mL Calcein AM Viability Dye (Fisher Scientific 15550597, Waltham, MA, USA) and 50 μg/mL propidium iodide (Fisher Scientific P21493) in 1X PBS solution (Dutscher P04-53500) was added into each well to evaluate cell viability. The plate was then incubated for 20 min at 37 °C, 5% CO_2_. The fluorescence of Calcein AM [excitation 469 nm, emission 525 nm] and propidium iodide [excitation 586 nm, emission 647 nm] was measured using a plate reader (Biotek Cytation 1, Winooski, VT, USA). Automatic analysis delivered numbers of living cells (Calcein count), numbers of dead cells (propidium iodide count), and viability (defined as the ratio of living cells to total cells). The results are expressed as the percentage of activation of hyaluronic acid synthesis normalized to cell viability. After normality and variance homogeneity were checked, data were analyzed with Brown–Forsythe and Welch ANOVA tests with Dunnett’s correction using PRISM 10.6.0(890) (GraphPad software, Boston, MA, USA). The statistical significance threshold was set at *p* < 0.05.

#### 4.2.3. Pro-Collagen I Production in Dermal Fibroblast Culture

NHDF isolated from human dermis (24-, 30- and 37-year-old female donors undergoing plastic surgery and having given informed consent; ethical committee reviews were not required by local regulations (France) for this type of tissue sourcing) were maintained in a specific medium composed of Dulbecco’s Modified Eagle Medium (DMEM) (Dutscher L0060-500, Bernolsheim, France) containing 10% serum (Sigma F7524, Burlington, MA, USA) and 1% antibiotics (penicillin/streptomycin, Sigma P0781) at 37 °C under 5% CO_2_ and 95% humidity. 25,000 cells/well were seeded in 96-well microplates in complete DMEM for 24 h. The culture medium was then replaced with serum-free DMEM for an additional 24 h to promote a quiescent cell state. Then, 25 µg/mL L-ascorbic acid (Sigma 95210), 0.08% high-MW HA (Phylcare Sodium Hyaluronate HW Plus, Lehvoss, Hamburg, Germany), or *Porphyridium* extract at 0.025% or 0.10% were added for an additional 24 h, alongside an untreated control. At the end of the incubation period, supernatants were collected for ELISA (Bio-Techne R&D Systems DY6220, Minneapolis, MN, USA), using the antibody supplied (R&D Systems DY6220) and following the supplier’s instructions. Briefly, 60 μL of a mixture of 1 μg/mL Calcein AM Viability Dye (Fisher Scientific 15550597, Waltham, MA, USA) and 50 μg/mL propidium iodide (Fisher Scientific P21493) in 1X PBS solution (Dutscher P04-53500) was added into each well to evaluate cell viability. The plate was then incubated for 20 min at 37 °C, 5% CO_2_. The fluorescence of Calcein AM [excitation 469 nm, emission 525 nm] and propidium iodide [excitation 586 nm, emission 647 nm] was measured using a plate reader (Biotek Cytation 1, Winooski, VT, USA). Automatic analysis delivered numbers of living cells (Calcein count), numbers of dead cells (propidium iodide count), and viability (defined as the ratio of living cells to total cells). The results are expressed as the percentage of activation of pro-collagen I synthesis normalized to cell viability. Data were analyzed through a Kruskal-Wallis test with Dunn’s correction using PRISM 10.6.0(890) (GraphPad software, Boston, MA, USA). The statistical significance threshold was set at *p* < 0.05.

#### 4.2.4. Collagen III Production in Dermal Fibroblast Culture

NHDF isolated from human dermis (35- and 37-year-old female donors undergoing plastic surgery and having given informed consent; ethical committee reviews were not required by local regulations (France) for this type of tissue sourcing) were maintained in a specific medium composed of Dulbecco’s Modified Eagle Medium (DMEM) (Dutscher L0060-500, Bernolsheim, France) containing 10% serum (Sigma F7524, Burlington, MA, USA) and 1% antibiotics (penicillin/streptomycin, Sigma P0781) at 37 °C under 5% CO_2_ and 95% humidity. 30,000 cells/well were seeded in 4-well slides in complete DMEM for 48 h. The culture medium was then replaced with serum-free DMEM, and 20 ng/mL TGFβ (Sigma T7039) or 0.10% *Porphyridium* extract was added for an additional 72 h, alongside an untreated control.

Cells were then fixed with AFA (MM France F/40877-36, Brignais, France) over 30 min at room temperature. Wells were rinsed with PBS, and cells were blocked in a solution comprising PBS, 3% BSA (Sigma A4503), and 0.5% TritonX100 (Fisher Scientific, 10102913), for 40 min at 37 °C. The cells were then rinsed with PBS and incubated with a rabbit monoclonal anti-collagen III antibody (Abcam ab184993) at 1:100 with PBS and 3% BSA overnight at 4 °C. After rinsing in PBS, the slides were incubated with goat anti-rabbit antibody (Alexa Fluor™ 568 at 1:1000, Invitrogen A11011) with PBS and 3% BSA for 2 h at room temperature. To visualize nuclei, the slides were incubated with Hoechst 1/5000 (Invitrogen H3570) with PBS for 5 min at room temperature. The slides were mounted with an aqueous fluorescence mounting medium (DAKO Agilent S302380-2, Glostrup, Denmark). Then, 5 images/well were acquired using a Nikon Instruments Eclipse Ti2 microscope (Tokyo, Japan) with a 20X objective. Collagen III fluorescence intensity and nuclei count were then quantified with the Image J 1.54P software [78]. Raw fluorescence intensity was normalized by cell count. After normality and variance homogeneity check, data were analyzed via Brown–Forsythe and Welch ANOVA tests using PRISM 10.6.0(890) (GraphPad software, Boston, MA, USA). The statistical significance threshold was set at *p* < 0.05.

#### 4.2.5. Anti-Inflammatory Effect

NHDF isolated from human dermis (24-, 30-, 35- and 37-year-old female donors undergoing plastic surgery and having given informed consent; ethical committee reviews were not required by local regulations (France) for this type of tissue sourcing) were maintained in a specific medium composed of Dulbecco’s Modified Eagle Medium (DMEM) (Dutscher L0060-500, Bernolsheim, France) containing 10% serum (Sigma F7524, Burlington, MA, USA) and 1% antibiotics (penicillin/streptomycin, Sigma P0781) at 37 °C under 5% CO_2_ and 95% humidity. 21,000 cells/well were seeded in 96-well microplates in complete DMEM for 24 h. Cells were then treated for 24 h with 0.002 ng/mL IL-1α (Sigma I2778) to induce an inflammatory response, either alone (untreated, induced control) or in the presence of 0.10% *Porphyridium* extract, 0.025 g/mL hydrocortisone (Sigma H0396) as positive control, or 0.02% low-MW HA (Phylcare Sodium Hyaluronate Extra LW, Lehvoss, Hamburg, Germany) as a benchmark. An untreated, uninduced negative control was carried out in parallel.

Following the 24-h incubation period, supernatants were collected for ELISA (Bio-Techne R&D Systems DY208, Minneapolis, MN, USA). Then, 60 μL of a mixture of 1 μg/mL Calcein AM Viability Dye (Fisher Scientific 15550597, Waltham, MA, USA) and 50 μg/mL propidium iodide (Fisher Scientific P21493) in 1X PBS solution (Dutscher P04-53500) was added to each well to evaluate cell viability. The plate was then incubated for 20 min at 37 °C and 5% CO_2_. The fluorescence of Calcein AM [excitation 469 nm, emission 525 nm] and propidium iodide [excitation 586 nm, emission 647 nm] was measured using a plate reader (Biotek Cytation 1, Winooski, VT, USA). Automatic analysis delivered the number of living cells (Calcein count), the number of dead cells (propidium iodide count), and viability (defined as the ratio of living cells to total cells). The results are expressed as the percentage of IL8 production normalized to cell viability. After normality and variance homogeneity were checked, data were analyzed via Kruskal–Wallis ANOVA analysis using PRISM 10.6.0(890) (GraphPad software, Boston, MA, USA). The statistical significance threshold was set at *p* < 0.05.

#### 4.2.6. Autophagy Activation in Aged Dermal Fibroblast Culture

Human primary fibroblasts were obtained from two female donors aged 67 and 30 years (respectively ‘aged’ and ‘young’ cells), undergoing plastic surgery and having given informed consent; ethical committee reviews were not required by local regulations (France) for this type of tissue sourcing.

##### Evaluation of Autophagy Flux

Fibroblasts were grown in DMEM (with pyruvate, glutamax, 4.5 g/L glucose; Thermo-Fisher GIBCO 31966-021, Waltham, MA, USA) supplemented with 10% FBS (Fetal bovine serum, Thermo-Fisher GIBCO 10500-064) and 1% PS (Penicillin-streptomycin, Thermo-Fisher 15140122). Cultures were maintained in an incubator equilibrated with 5% CO_2_ at 37 °C.

An Autophagy Detection Kit (Abcam ab139484, Cambridge, UK) was used to detect autophagy in live cells by fluorescence microscopy. Its 488-nm excitable green fluorescent detection reagent becomes brightly fluorescent in autophagy vesicles. A nuclear counterstain (blue) was used to highlight cellular nuclei.

Aged fibroblasts were treated for 24 h with 0.1% *Porphyridium* extract in culture medium. Following incubation, the medium was removed, and the cells were washed twice with Assay Buffer. Cells were covered in assay buffer, incubated for 30 min at 37 °C, and washed again. The cells were fixed with 10% formalin over 15 min, then triple-washed. PBS+/+/glycerol 50/50 was added to the plate wells. Young fibroblasts were included as a positive control, as was an untreated aged fibroblast leg.

Microplates were observed with an epifluorescence microscope (Zeiss, Axio Imager Z1, ApoTome, Zen2 blue edition software, Oberkochen, Germany) equipped with a DAPI channel and a GFP channel to generate images, which were then analyzed (ImageJ/FIJI 1.53 software [103]). The results were expressed as total fluorescent intensity normalized to the number of nuclei, and compared to the ‘young’ fibroblast control. 

Statistical analysis was performed using the Wilcoxon test using PRISM 10.6.0(890) (GraphPad software, Boston, MA, USA). The statistical significance threshold was set at *p* < 0.05.

##### Effect of the Extract on Autophagy-Related Gene Transcription

Aged human primary dermal fibroblasts from the 67-year-old donor above were grown in DMEM (Dulbecco’s Modified Eagle Medium with pyruvate, glutamax, 4.5 g/L glucose; GIBCO 31966-021) supplemented with 10% FBS (Fetal Bovine Serum, GIBCO 10500-064) and 1% PS (Penicillin-streptomycin, Thermo-Fisher 15140122). 

The fibroblasts were treated with 0.1% *Porphyridium* extract in medium for 12 h, opposite an untreated control leg.

Total RNA was extracted and purified using an RNeasy Mini Kit (Qiagen 74136, Venlo, The Netherlands), following the manufacturer’s instructions. The quality controls and total RNA quantification were performed with an Agilent (Santa Clara, CA, USA) RNA Nano kit using the Agilent 2100 bioanalyzer and Nanodrop spectrophotometer (Thermo-Fisher). 

Total RNA was reverse-transcribed with the Superscript VILO cDNA Synthesis Kit (Invitrogen 11754-050, Thermo-Fisher) according to the manufacturer’s instructions. Quantitative PCR (qPCR) was performed using a Platinum SYBR Green qPCR SuperMix-UDG Kit (Invitrogen 11733-046, Thermo-Fisher) according to the manufacturer’s instructions, and CFX-connect (Bio-Rad, Hercules, CA, USA). All qPCR experiments were performed in duplicate. The primer sequences were as follows (forward/reverse, respectively, in each case): ATG7 (NCBI #10533): ATGATCCCTGTAACTTAGCCCA/CACGGAAGCAAACAACTTCAAC; ATG8 (NCBI #11345): AGGTCTCAGGCTCTCAGATTG/ACAGGAAGATCGCCTTTTCAGA; DNM1L (NCBI #10059): TTTGACACTTGTGGATTTGCCA/AGTGACAGCGAGGATAATGGA; LAMP2A (NCBI #3920): TGGCAATGATACTTGTCTGCTG/ACGGAGCCATTAACCAAATACAT; EPHA2 (NCBI #1969): TGGCTCACACACCCGTATG/GTCGCCAGACATCACGTTG; and housekeeping gene GAPDH (NCBI #2597): GGAGCGAGATCCCTCCAAAAT/GGCTGTTGTCATACTTCTCATGG. The PCR program ran as follows: 50 °C for 2 min; then 95 °C for 2 min; followed by 39 cycles of [95 °C for 15 s and 60 °C for 30 s/62 °C for 30 s for EPHA2]; and finally 60 °C for 5 s and 95 °C for 5 s.

For each study leg, three biological replicates were carried out, and each sample was analyzed in technical duplicate for every gene. A ΔΔCt analysis normalized to GAPDH was performed: For each sample, the mean Ct value was calculated from the technical duplicate wells. Then, ΔCt was obtained by subtracting the Ctmean of the housekeeping gene (GAPDH) from the Ctmean of the gene of interest. For each study leg, ΔΔCt was calculated as the ΔCt of each sample minus the mean ΔCt of the three replicates for the untreated control. Relative expression (QR) was computed as 2^−ΔΔCt^. For each study leg, the final reported value corresponds to the mean QR of the three biological replicates. QR data were analyzed via two-tailed unpaired Student’s *t*-test using PRISM 10.6.0(890) (GraphPad software, Boston, MA, USA). The statistical significance threshold was set at *p* < 0.05. 

### 4.3. Testing Methods—Clinical

#### 4.3.1. General Design

A total of 90 subjects were recruited (in 3 groups of 30), aged 35 to 65 years, all female, of all skin types, and all selected for having a history of hyaluronic acid filler use; all types of skin were included. A phototype (Fitzpatrick) range of I to IV was selected, and the ethnic distribution included ca. 2/3 Caucasian subjects and 1/3 Brazilian (i.e., mixed-African) subjects per group (if possible, volunteers of Asian ethnicity were to be included on an opportunistic basis). Menopausal status was recorded.

The study was performed according to the Declaration of Helsinki principles and subsequent amendments, and in the spirit of Good Clinical Practice Guidelines and general principles of Portugal Law 46/2004 of 19 August 2004. The protocol and test conditions were reviewed by the Review Board of Ph.D. Trials^®^ Portugal (opinion nº 10107/2023, 10108/2023 and 10109/2023, approval date 2 December 2022; RNEC submission number 489243). Study participants gave informed consent in writing.

This study was run following a double-blind, randomized, placebo-controlled design, in three groups (Placebo, Benchmark, and Active). Product formulations are detailed in Appendix B. The Active leg contained 1% of the Porphyridium extract, while the Benchmark leg contained a total of 1% HA (0.2% low molecular weight and 0.8% high molecular weight, reflecting a common practice in the cosmetic industry). The product was applied on the face twice a day for 28 days. The trial’s timeline was designed to demonstrate the *Porphyridium* extract’s immediate and short-term effects, and conformed to current standard practices in the cosmetic industry: measurements were carried out at D0, before and immediately (1 h) after product application, as well as at D1 and D28 (without product application).

The amount of product consumed by each panelist over the course of the study was evaluated by weighing the product dispenser at D0 and D28, indicating that the panelists used very similar and consistent product amounts across the three groups: the mean consumption per application was 1.31 g ± 0.37 g for the placebo formulation; 1.33 g ± 0.37 g for the benchmark formulation; and 1.29 g ± 0.40 g for the active formulation containing 1% of *Porphyridium* extract.

All evaluations were performed in one of two time periods (9 h 00–13 h 00 and 13 h 00–19 h 00), so as to minimize circadian variations, in a climate-controlled room and after at least 15 min of acclimatization (T = 21 °C ± 2 °C; RH = 55% ± 10%).

#### 4.3.2. Measurements

##### Skin Plumpness, Wrinkling, Roughness, Radiance, and Spots

Skin plumpness, wrinkling, roughness, and spots were evaluated using a Visia-CR device with PRIMOS (Canfield, Parsippany, NJ, USA). Standardized photographic images of both hemifaces were obtained before, during and after the treatment (D0, D7 and D28) with normal, cross-polarized, and parallel-polarized lighting (the latter for fringe projection analysis only), in order to quantify the evolution of wrinkles and spots (visible, red, brown, and UV). A mask is created for the defined measurement area, and applied in subsequent images in order to calculate differences vs. D0. Visia images were also used as the source for illustrative macrophotography records. Skin radiance was analyzed through the difference in white (glow) pixels between cross-polarized and parallel-polarized images. Skin color is expressed in L, a, b Hunter color space parameters based on the analysis of the Visia images. The integrated PRIMOS system evaluated wrinkles and texture (roughness) through deviation analysis of fringe projection images, expressed in the following indices: Wrinkle count, Wrinkle volume, Wrinkle average depth, Ra = arithmetic mean of the skin surface, and Rz = mean of the 5 biggest peaks and the 5 lowest valleys in the image area. Finally, skin plumping (or conversely, sagging) is evaluated as the difference in 3D volume of the measurement area between a given measurement point and D0.

##### Skin Density

Skin density was evaluated by ultrasonography. Ultrasound images were generated based on echo from dense tissue, using a Dermascan C ultrasound system (Cortex technology, Aalborg, Denmark) with a 20-MHz ultrasound probe. SLEB (Sub-epidermal Low echogenic Band) dermal density was calculated. 

##### Skin Barrier Function

Skin barrier function, as Transepidermal Water Loss (TEWL), was assessed using a Tewameter™ (Courage & Khazaka, Cologne, Germany), measuring water diffusion flux, which expresses the amount of water transported by surface unit for a determined period of time. The measurement was carried out in the malar area. 

##### Skin Biomechanics by Cutometer

Skin biomechanical evaluation was performed using a Cutometer^®^ dual MPA 580 with a 2-mm probe (Courage & Khazaka, Koln, Germany), which measures the mechanical deformation of the upper skin layers under negative pressure (suction). The penetration depth of tissue into the probe (indicating the extent of skin deformation) is determined by an optical measurement system. This initial deformation indicates skin firmness. The skin’s elasticity is evaluated through its ability to return to its original position when the suction is discontinued.

##### Clinical Evaluation by Trained Dermatologist

Grading of skin wrinkling at the corner of the eye and nasolabial fold was carried out by trained clinicians, based on Bazin’s scale [104].

Clinicians also graded skin roughness on the whole face, according to a five-point scale, from Score 0: no roughness, perfectly smooth and supple skin; to Score 4: extreme roughness, very significant irregularity and major perturbation of the cutaneous relief. (Note: The scale ranges from perfect to severely flawed, meaning that a decrease in grade reflects an improvement in the skin condition.) 

#### 4.3.3. Statistical Analysis

Data analysis was performed using Prism 9 (GraphPad, Boston, MA, USA). The statistical significance threshold was set at *p* < 0.05, following general practice in the art. Statistical tests were selected on the basis of normality tests: a parametric test (unpaired Student’s *t*-test) was applied when the normality was positive, and a non-parametric test (Mann-Whitney test) was used when the normality was negative. 

## Data Availability

The data presented in this study are available upon request from the corresponding author.

## Appendix A

Table A1 presents a clear gel formulation used in evaluating the formulability and formula stability of the *Porphyridium* extract. Table A2 similarly presents a model oil-in-water formulation used for the same purposes.

**Table A1:** Model clear gel formulation.

Ingredient INCI Name	% in Control	% in Active
Water	QS 100%	QS 100%
Chlorphenesin	0.30	0.30
Phenoxyethanol	0.80	0.80
Acrylates/C10-30 alkyl acrylate copolymer	0.30	0.30
Water and Sodium hydroxide (33%)	QS for pH 5.0–5.5	QS for pH 5.0–5.5
*Porphyridium cruentum* extract	0.00	2.00
TOTAL	100.00	100.00

**Table A2:** Model oil-in-water emulsion formulation.

Ingredient INCI Name	% in Control	% in Active
Water	QS 100%	QS 100%
Xanthan gum	0.50	0.50
Glyceryl Stearate Citrate	2.00	2.00
Polyglyceryl-3-Stearate	2.00	2.00
Prunus Amygdalus Dulcis (Sweet Almond) Oil	3.00	3.00
Cetyl Alcohol	2.00	2.00
Butyrospermum Parkii (Shea) Butter	3.00	3.00
Coco-Caprylate	3.00	3.00
Tocopherol (and) Helianthus Annuus (Sunflower) Seed Oil	0.20	0.20
Water (and) Sodium Levulinate (and) Sodium Anisate	4.00	4.00
*Porphyridium cruentum* extract	0.00	2.00
TOTAL	100.00	100.00

## Appendix B

Table A3 presents the formulations used in the clinical trial.

The following HA components were used in the benchmark formulation: low-molecular-weight HA—Phylcare Sodium Hyaluronate Extra LW; high-molecular-weight HA—Phylcare Sodium Hyaluronate HW Plus (Lehvoss, Hamburg, Germany).

**Table A3:** Clinical trial formulations.

Ingredient INCI Name	% in Placebo	% in Benchmark	% in Active
Water	98.80	97.80	97.80
Chlorphenesin	0.30	0.30	0.30
Phenoxyethanol	0.60	0.60	0.60
Acrylates/C10-30 alkyl acrylate copolymer	0.30	0.30	0.30
Water and Sodium hydroxide (33%)	QS	QS	QS
Sodium hyaluronate (low MW)	0.00	0.20	0.00
Sodium hyaluronate (high MW)	0.00	0.80	0.00
*Porphyridium cruentum* extract	0.00	0.00	1.00
TOTAL	100.00	100.00	100.00
